# Wild geladas (*Theropithecus gelada*) in crops—more than in pasture areas—reduce aggression and affiliation

**DOI:** 10.1007/s10329-021-00916-8

**Published:** 2021-06-01

**Authors:** Marta Caselli, Anna Zanoli, Carlo Dagradi, Alessandro Gallo, Dereje Yazezew, Abebe Tadesse, Michele Capasso, Davide Ianniello, Laura Rinaldi, Elisabetta Palagi, Ivan Norscia

**Affiliations:** 1 Department of Life Sciences and Systems Biology, University of Torino, via Accademia Albertina 13, 10123 Torino, Italy; 2 Department of Biology, Debre Behran University, Debre Berhan, Ethiopia; 3 Department of Veterinary Medicine and Animal Production, University of Napoli Federico II, Naples, Italy; 4 Unit of Ethology, Department of Biology, University of Pisa, via Volta 6, 56126 Pisa, Italy; 5 University of Pisa, Natural History Museum, Pisa, Italy

**Keywords:** Primates, Behavioral change, Social behavior, Human impact, Primate health, Ethiopia

## Abstract

Human–primate interfaces are expanding and, despite recent studies on primates from peri-urban environments, little research exists on the impact of agriculture and/or pasture areas on primate social behavior and health. We assessed how crop/pasture areas potentially alter social behavior and health of wild geladas (*Theropithecus gelada*) frequenting the unprotected area of Kundi (Ethiopia). We predicted that compared to pasture areas, crop areas (i) would be more challenging for geladas (prediction 1) and (ii) would have a greater impact on both aggressive and affiliative behavior, by reducing grooming time and enhancing competition (prediction 2). During January–May 2019 and December 2019–February 2020, we collected data (via scan, focal animal sampling, and video analyses) on direct human disturbance, external signs of pathologies and social behavior of 140 individuals from 14 one-male units and two all-male units. Animals experienced the highest level of human disturbance in crop areas (in line with prediction 1). Individuals from the groups preferentially frequenting crop areas showed the highest prevalence of external signs of pathologies consistent with chemical and biological contamination (alopecia/abnormally swollen parts). We collected 48 fecal samples. Samples from frequent crop users contained the highest rates of parasitic elements/gram (egg/larva/oocyst/cyst) from *Entamoeba histolytica/dispar*, a parasite common in human settlements of the Amhara region. In crop areas, subjects spent less time grooming but engaged in lower rates of intense aggression (in partial agreement with prediction 2). We speculate that the reduction in social behavior may be a tactic adopted by geladas to minimize the likelihood of detection and maximize food intake while foraging in crops.

**Supplementary Information:**

The online version contains supplementary material available at 10.1007/s10329-021-00916-8.

## Introduction

The growing expansion of human settlement (Koh and Wilcove 2008) is causing changes in wildlife behavior due to a forced coexistence of wildlife and humans (Sih et al. 2011). Previous investigations report behavioral changes in different taxa (reptiles: Batabyal et al. 2017; birds: Blumstein et al. 2005; mammals: Belton et al. 2018). Nonhuman primates (hereafter primates) are no exception and are particularly affected because approximately 30% of the existing species live in proximity to human settlements and rely on anthropic land cover for their maintenance activities (McLennan et al. 2017; Galán-Acedo et al. 2019).

Various types of human–primate interfaces, including tourist-provisioned sites, temples, urban settlements, and agricultural fields (Kaburu et al. 2019; Balasubramaniam et al. 2020; Jaman and Huffman 2013), are described in the literature. Agricultural areas can have a particularly strong impact on primate behavior (Arroyo-Rodríguez and Fahrig 2014) because crops are often associated with close human settlements (Minta et al. 2018). They can include patches with clumped, high-quality and palatable resources, leading to high-risk crop foraging by primates (Riley et al. 2013). Hill (2018) proposed two hypotheses to explain crop foraging: the *crops as fallback foods hypothesis*, according to which primates would feed on crops when wild resources are scarce, and the *crop foraging as an optimizing strategy hypothesis*, according to which the high risk associated with crop foraging would be compensated by an increase in nutritional intake, with consequent benefits for reproductive potential.

One of the main risks that primates face when frequenting areas in which humans are present, including agricultural fields, is related to direct or indirect pathogen transmission among humans, livestock, and primates (Goldberg et al. 2007; Krief et al. 2010). Such transmission can include gastrointestinal parasites, such as protozoans in *Gorilla gorilla gorilla* (*Giardia intestinalis*; Sak et al. 2013), several nematode species in *Papio* spp. (Hahn et al. 2003), and, if wild or domestic canids are present, the cestode *Taenia serialis* in *Theropithecus gelada* and other primates (Schneider-Crease et al 2017; Chanove et al. 2019).

The health of wild primates can also be impacted when their home ranges include agriculture land and herbicides and other chemical pollutants are used on crop fields (Garabrant and Philbert 2002). For example, 2,4-dichlorophenoxyacetic acid, frequently used for weed control (de Castro Marcato et al. 2017), has been associated with the presence of alopecia (e.g. in dogs, Charles et al. 1996), tumors (in humans, Anthony and Saleh 2013), and reproductive problems (e.g. in chimpanzees and olive baboon, Krief et al. 2017). We urgently need more evidence on the possible harm due to the ingestion of herbicides and pesticides.

Finally, different types of human–primate interfaces may variably influence primate social behavior. Chowdhury et al. (2020) found that in chacma baboons, *Papio ursinus*, social grooming decreased in anthropogenic areas. Other studies were mostly focused on macaques. For example, in peri-urban areas, *Macaca radiata* showed reduced grooming effort due to interaction with both visitors and local residents (Balasubramaniam et al. 2020). In temple areas, depending on the level of human–monkey interaction, *Macaca mulatta* can reduce social grooming considerably (Kaburu et al. 2019), but in urban areas they can increase grooming and play compared to rural areas (Jaman and Huffman 2013)*.* The time spent grooming in *Macaca fascicularis* varies depending on whether the interaction with humans is moderate or high (Marty et al. 2019).

The social behavior of primate groups frequenting agricultural lands may be particularly affected for at least three reasons. First, the measures used by humans to protect their crops, such as chasing, throwing objects, or even shooting at animals (Osborn and Hill 2005), can disrupt primate behavior (McKinney 2015; McLennan et al. 2017). Second, the high-quality, concentrated resources found in agricultural lands can lead to reduced affiliation and increased overt competition (Jaman and Huffman 2013; Arseneau-Robar et al. 2016). Third, time budget trade-offs can come into play, as in agricultural areas primates might be constrained by time linked to a higher risk of being herded by humans that monitor them to keep them away (Priston et al. 2012; Chowdhury et al., 2020).

Based on this framework, our goal was to contribute to a better understanding of how different human–primate interfaces can affect the health and social behavior of nonhuman primates. Specifically, we investigated whether the relative use of two different human–primate interfaces, namely agriculture and pasture, affected the health and the social behavior of a population of wild geladas (*Theropithecus gelada*), a primate species endemic to Ethiopia. Geladas are group-living, terrestrial, and mostly herbivorous; consequently, part of their natural plant food species is shared with livestock (Fashing et al. 2014). Moreover, the products of cultivated plants (e.g. *Eragrostis tef*) are also highly attractive to geladas, which can approach human settlements and enter crop fields in search of food (Abu et al. 2018). Based on the observation that primates frequenting crops can be exposed to direct (e.g. active chasing: Osborn and Hill 2005) and indirect human disturbance (e.g. chemical and biological sources of potential pathology: Garabrant and Philbert 2002; Nunn et al. 2006), we predicted that the geladas using the crop area the most would be exposed to more frequent direct human disturbance (prediction 1a), higher risk of developing pathologies (prediction 1b), and increased risk of infection by parasites typical of human settlements (prediction 1c).

Geladas live in a multi-level society whose basic unit is the one-male/multi-female unit (hereafter, OMU) (Dunbar and Dunbar 1975; Zinner et al. 2018). An OMU generally comprises one adult male, several adult females, and their offspring. Bachelor groups, separate from OMUs, are called all-male units (hereafter, AMU). OMUs and AMUs can form teams, bands and, at a larger level, herds, which can include hundreds of individuals (Dunbar and Dunbar 1975; Snyder-Mackler et al. 2012; Zinner et al. 2018). High-intensity sporadic aggression is observed when a male tries to take over a group or to claim a territory (Beehner and Bergman 2008). However, the absence of a strict reproductive season and the control of a single male over a group of females largely reduces inter-male competition over females (Dunbar and Dunbar 1975). Moreover, groups are characterized by extremely high tolerance levels (Dunbar and Dunbar 1975). As a result, gelada societies are characterized by low rates of inter- and intra-group (OMUs/AMUs) aggression and high levels of affiliative social grooming between group members (Dunbar and Dunbar 1975; Mancini and Palagi 2009). Because human interference and resource competition in primates can lead to decreased affiliation (Jaman and Huffman 2013) and increased aggressive patterns (Arseneau-Robar et al. 2016; Thatcher et al. 2019), both of which can jeopardize group cohesion and social stability, we predicted that geladas would spend less time grooming (prediction 2a) and engage in aggression of higher intensity when in the crop area compared to the pasture area (prediction 2b).

## Methods

### Study site and subjects

This study was conducted with a population of wild geladas frequenting the Kundi plateau, in the Wof-Washa area (Ethiopia, Amhara region, N9°40.402’ E39°45.060’; altitude (min–max): 3370–3592 m). We followed the subjects from January to May 2019 and from December 2019 to February 2020, spanning the dry and the beginning of the small rainy season (for further information see Appendix S1), on a daily basis, five days per week (excluding days with heavy rain or mist), from around 9:30 to 17:00 (for a total of 94 full days and a total of 658 h). We considered that the small rainy season (cf. Yazezew et al. 2020) had started when the rain set in for three consecutive days. The late dry and early wet periods—often including the post-harvesting phase—can be key periods of nutritional need, possibly associated with crop raiding by geladas searching for crop food remains and seeds (Hirvonen et al. 2016; Dunbar 1977).

Surrounded by cliffs, the Kundi plateau (26 ha) is characterized by crop (about 12 ha) and pasture areas (about 14 ha), which have the same visibility conditions (Fig. S1). In this study, we defined “crop area” as the agriculture fields (including human settlements) and the zone within 300 linear meters from the closest house or cultivated land. This criterion allowed for cultivated land, houses, domestic animal shelters, and passage zones from crop to crop or from crop to houses to be included in the “crop area.” We defined “pasture area” as the grassland without human settlements and cultivated fields, where livestock (horses, goats, sheep, donkeys, and cows) grazed during the day, led by shepherds. During the study period, animals spent 77.083 ± 14.360 (mean ± SE) and 276.458 ± 23.500 (mean ± SE) non-consecutive minutes per day in the crop and pasture areas, respectively. Gelada groups were free to move down the cliffs from the plateau. Further information on the study is available in Appendix S1.

In the first month of the study, a subset of groups frequenting the Kundi plateau were habituated and surveyed by four to six researchers (EP, IN, MaC, AZ, CD, AG). Group size, sex ratio, age ratio, and natural markers of the central male and/or other individuals (as detailed below) were used to identify gelada groups (one-male unit; OMU/all-male unit; AMU), based on Dunbar and Dunbar (1975) criteria. This process required around one month and was facilitated by video-recording of the groups. We were able to survey 14 OMUs and two AMUs and counted 27 adult males, 79 adult females, 60 subadult individuals, 35 juveniles, and 65 infants (31 late, 21 early, 13 black; further information on the population is available in Appendix S1). The number of groups present on the plateau on a daily basis was 8.706 ± SE 0.950 (mean ± SE).

Individual discrimination was achieved for 140 subjects (excluding infants) by considering long-lasting distinctive features (including sex, size, permanent scars, deformations, and particular shapes of the red chest area in adults; Dunbar and Dunbar 1975). Such features were identified during field observations or via video recordings during and after the field data collection.

### Field data collection

Each day four observers (MaC, AZ, CD, AG) went on the Kundi plateau and split into two groups to search for the gelada groups toward the top and the bottom of the plateau, respectively. The group composition of observers changed every week, following a rotation schedule. One observer (videographer) recorded the videos and the other assisted the videographer by vocally recording the ongoing activities and the subjects involved in the behavior. Not all of the identified gelada groups were present on the highland every day. Thus, on each day (after the end of the habituation period) data were collected on the visible and recognizable groups, giving priority to the less commonly observed groups when multiple groups were present to reduce observation imbalance and ensure sufficient data collection for all groups.

We conducted scan sampling (Altmann 1974) live (not on video) at 10-min intervals on the recognized, visible groups present on the plateau each day. We gathered a mean of 304.357 ± SE 43.879 scans per group covering the whole daily observation period. Multiple groups could be present in a scan. Whenever possible, we recorded for the purpose of this study (i) group identity, (ii) GPS position based on the central male position (Garmin GPS Map 64), and (iii) the percentage of individuals foraging.

Data on direct human–gelada interactions (e.g. chasing animals, throwing stones, sticks; see table S1 for a detailed description, video MPEG-1) were collected via an all-occurrences sampling method (Altmann 1974) to gather data on each possible episode.

On the recognizable groups, we also collected data via two video cameras (Panasonic HC-V180, full-HD, 50 fps, optical zoom 50x) for a total of 120 h of videos. We gathered a mean of 8.071 ± SE 1.336 video hours per group and a mean of 2.128 ± SE 0.198 video hours per subject, spreading the observational effort across morning and afternoon.

Grooming videos were collected via 10-min focal sampling (Altmann 1974), with the focal subject being selected on the basis of the criteria explained above (giving priority to visible, recognizable, and less observed subjects). If the grooming continued, the recording went on until the end of the grooming session to allow analyses on grooming duration. This rule was applied to all dyads, and extra video duration (after 10 min) was considered only to calculate grooming duration (normalized as explained in the behavioral data section). The videos including grooming lasted on average 11.502 ± SE 0.686 min and involved 22 adult males (belonging to both OMUs and AMUs), 30 adult females, 5 immature males, and 2 immature females.

Owing to the tolerant nature of the study species, aggressive encounters are known to be infrequent (Bergman 2010; Dunbar 2014). Hence, data on aggressive events were collected via all-occurrences sampling (Altmann 1974). Cameras were always kept on, on the clearly visible groups. While the videographer recorded the scene, the assistant would describe the aggressive event aloud to also gather data on what happened off-screen if necessary. At least three aggressive events per group were recorded, involving 23 adult males, 61 adult females, 29 immature males, and 10 immature females. The observed aggressions occurred to displace individuals from a foraging spot.

### Health and disturbance data, and operational definitions

We calculated how frequently the OMUs + AMUs (*N* = 16) were present in the crop area by considering the number of scans in which each group was inside the crop area normalized over the total scans per group. The group position was assessed via GPS coordinates, referring to the alpha-males. We then separated the groups into two categories (“frequent crop users” and “infrequent crop users”), depending on whether the frequencies fell above or below the median frequency of the proportion of scans per group recorded in crops (median = 0.189; range = 0.020–0.340; Table S2) (Fig. S1).

Then, we considered the number of events of direct human disturbance (e.g. humans chasing geladas using stones, dogs, sticks, shooting; Table S1, Figure S2, video MPEG-1) for frequent and infrequent crop users, normalized over the total scans per group in each area (i.e. crop vs. pasture).

**Fig. 1:**
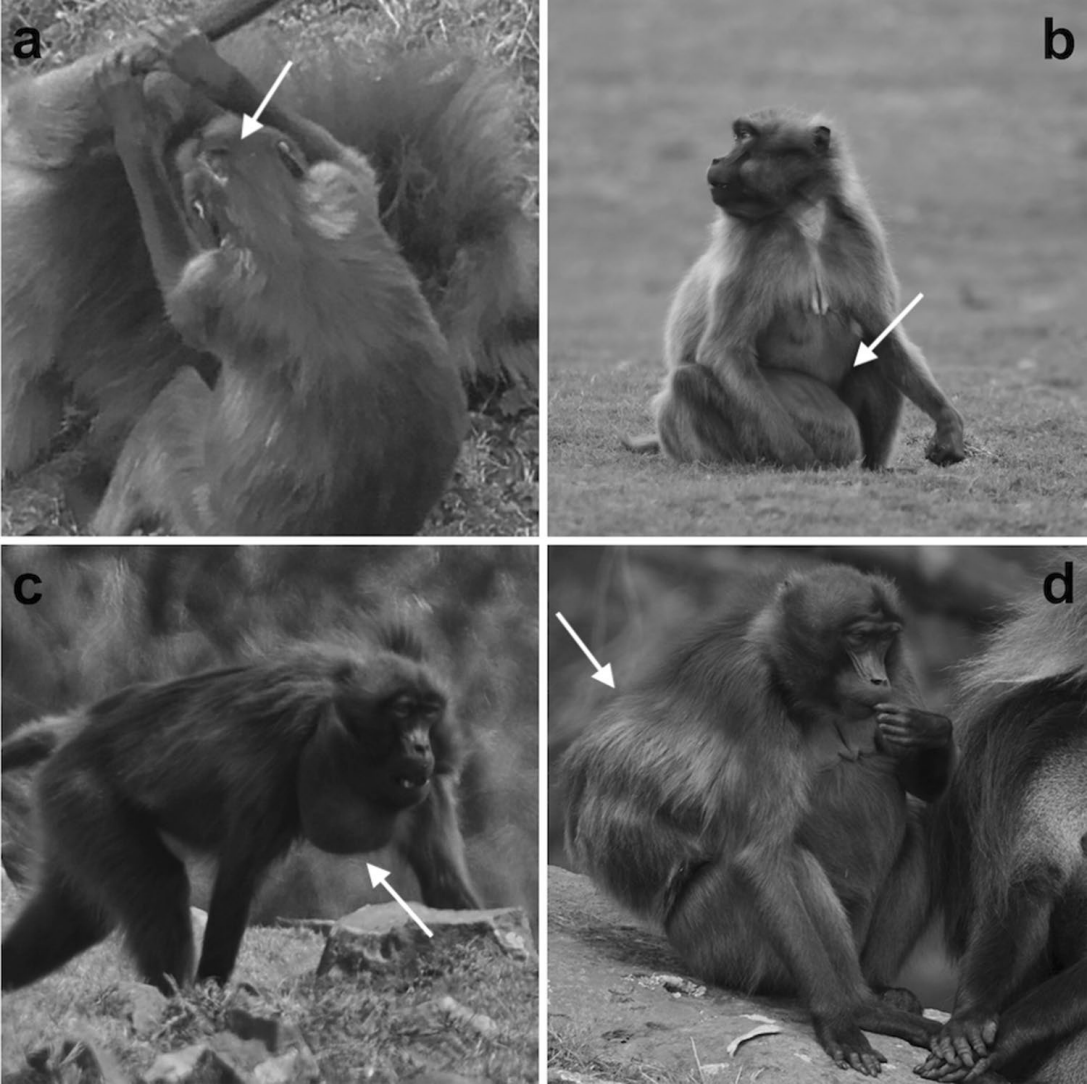
Pathologies observed in the geladas from the Kundi plateau: (**a**) adult female with alopecia, (**b**-**c**) adult female with abnormal swelling, (**d**) adult female with both alopecia and swelling. Photos by: Ivan Norscia, Alessandro Gallo, Carlo Dagradi

On the basis of photos and videos, the individuals (adults and immatures) were considered as bearing external signs of pathology when they showed at least one of the following external signs: abnormal swelling on trunk, limbs, and/or neck, probably related to *Taenia serialis* infection, as it has been found in other gelada populations (Ohsawa and Dunbar 1984; Nguyen et al. 2015; Schneider-Crease et al. 2017); and alopecia, defined as hair loss either diffuse or patchy, in areas where the loss could not be caused by infant clinging (Fig. 1). The external signs of pathologies were considered for males and two categories of females (lactating and non-lactating) due to the effect that lactation can have on the immune system (Wang 2016). Depending on the group they belonged to, individuals were assigned to either frequent or infrequent crop user groups. Descriptive statistics on the external signs of pathology are included in Appendix S1.

### Behavioral data and operational definitions

We determined the daily frequency of foraging in the pasture and crop areas by considering the number of scans in which at least 10% of animals were foraging in either area normalized on the total number of daily scans per area.

Data on grooming were extracted from videos using the focal animal sampling (Altmann 1974). To calculate grooming duration, we considered a grooming session as started when one of the two individuals began cleaning the fur of the other, and as finished when grooming was interrupted for at least 10 s (Mancini and Palagi 2009). We recorded (i) groomer and grooming receiver identities, (ii) age class of both individuals (adult or immature), (iii) sex class (male or female), (iv) time spent grooming, and (v) area where grooming took place (pasture or crop). Because the observation time varied across dyads, for each dyad we divided the daily time spent grooming by the focal daily observation time of that dyad (normalized data).

The aggressive events were extracted from video- and audio-recorded information, following an all-occurrences method (Altmann 1974) on the observable groups. For each aggressive event, we recorded the following data: (i) the identity of the aggressor (individual that initiated the first agonistic pattern) and the identity of the recipient (the individual that received the first aggressive pattern), (ii) age class (adult or immature), (iii) sex class (male or female), (iv) intensity of aggression, i.e. mild (chasing or chasing attempt without contact between opponents) or strong (chasing with contact between opponents; video MPEG-2), (v) whether aggression was intra- or inter-group, and (vi) the area where the aggression took place (pasture or crop). We recorded a total of 114 aggressive events, with a minimum of three aggressive events per group. All videos were analyzed via the free software VLC 3.0.6 (©VideoLAN) by MaC and AG (Cohen’s value for inter-observer reliability calculated on 10% of the total grooming/aggressive events ≥ 0.75).

### Fecal sample collection and parasitological analyses

We collected 48 fresh fecal samples (preserved in 10% formalin) from 48 unique individuals during observations and identified the samples as from individuals in the frequent or infrequent crop user group. The number of gastrointestinal parasitic elements (egg/larva/oocyst/cyst)/g of feces was determined using the FLOTAC pellet dual technique (Cringoli et al. 2010). This protocol is a multivalent, quali/quantitative copromicroscopic method for detecting parasitic elements (eggs, larvae, oocysts, and cysts) in animal fecal samples, with an analytical sensitivity of one parasitic element per gram of feces (EPG/LPG/OPG/CPG). The pellet technique is performed for samples with unknown fecal material weight, so the weight of the fecal material can be obtained after weighing the sediment in the tube (pellet) after filtration and centrifugation of the fecal sample. These steps are very important for discriminating between parasites and pseudoparasites, considering that the identification of parasites in fecal samples is often complicated by the high fiber content of the animal diet, as well as the common presence of pollen, plant tissue, flowers, and invertebrate fragments (accidentally ingested with the plants), all of which can be misclassified as parasitic structures (Alvarado-Villalobos et al. 2017).

Two different flotation solutions were used to detect the gastrointestinal parasites: FS2 (sodium chloride solution, specific gravity = 1200) and FS7 (zinc sulfate solution, specific gravity = 1350). Different magnifications were used, ×100 and ×400, respectively, for the study of egg/larvae of helminths and cysts/oocysts of protozoa.

The diagnostic technique described above does not allow the identification at the species/assemblage level, so it was not possible to measure the specific richness.

### Statistical analyses

Because of the small sample size (*N* < 10: *N*_frequent_OMU_crop_users_ = 8, *N*_infrequent_OMU_crop_users_ = 5; not testable for normality), we employed a nonparametric Mann–Whitney test (SPSS 20.0) to compare the frequencies of direct human disturbance (Table S1) to primates between frequent and infrequent crop users. We included in the analyses the groups that underwent at least two disturbance events (Table S1, Fig. S2, video MPEG-1). We excluded three groups not meeting this condition. Exact values were selected following Mundry and Fischer (1998).

Owing to non-normal variable distribution (Kolmogorov–Smirnov test: *N*_days_ = 48; *P* < 0.05), we used the nonparametric paired Wilcoxon signed-rank test (Siegel and Castellan 1988) to compare the daily frequency of foraging in crop and pasture areas. We applied a Monte Carlo randomization (10,000 permutations) (Bros and Cowell 1987) to account for possible data pseudoreplication (same individuals present on different days).

We ran three generalized linear mixed models (GLMM) with three different target (dependent) variables, on three different aspects: presence of external signs of pathology (GLMM_1_), grooming duration (GLMM_2_), and aggression intensity (GLMM_3_).

GLMM_1_ was run to explore what individual features could affect the presence of external signs of pathology. We included in the model the occurrence of external signs of pathology as a dependent, binomial variable (factorial; presence/absence). We included four predictors as fixed factors: age class (factorial; adult/immature, excluding infants), sex class according to the presence of infants under lactation (factorial; non-lactating females; lactating females; males), group category based on the level of frequenting the crop area (factorial; frequent and infrequent crop users), and the group size (numeric). The group identity was included as a random factor.

To compare the parasite load (number of parasitic elements/g of feces) between frequent and infrequent crop users, we applied the exact Mann–Whitney nonparametric test (Mundry and Fischer 1998; Siegel and Castellan 1988; non-normal distributions; Kolmogorov–Smirnov test: *N* = 48, *Ancylostomatidae* *P* = 0.001; *Chilomastix* spp. *P* < 0.001; *Entamoeba histolytica/dispar* *P* < 0.001; *Endolimax nana* *P* = 0.007; *Giardia intestinalis* *P* < 0.001). The level of probability was adjusted according to the Bonferroni correction (*α* = 0.010).

GLMM_2_ was run to test the effect of area (crop/pasture) on the daily time spent grooming by dyads. We included the following predictors (factorial fixed factors): area where grooming took place (pasture/crop), season (dry/small rainy), age class of the two subjects involved in the grooming (adult/immature), sex class (male/female), crop use frequency (frequent/infrequent crop users), and group type (OMU/AMU). The grooming dyad and the unit identity were included as random factors.

Finally, GLMM_3_ was run to investigate what variables could affect the intensity of aggression. Due to the small number of aggressive events involving AMU (*N* = 2), for this analysis we considered only aggressive events involving OMUs. The model included the intensity of aggression as a binomial, dependent variable (mild/strong). We included the following fixed factors: area where the aggression took place (pasture/crop), season (dry/small rainy), dyad age class (same/different), dyad sex class (same/different), dyad group (inter-/intra-group aggression), and crop use frequency of both aggressor and recipient (frequent/infrequent crop users). The aggressor–recipient dyad and the OMU membership of individuals were included as random factors.

We fit all three models in R (R Core Team 2018; version 3.5.1) using the function “glmer” (in the case of binomial, dependent variable) of the R package *lme4* (Bates et al. 2015). We established the significance of the full model by comparison to a null model comprising only the random effects (Forstmeier and Schielzeth 2011). We used a likelihood ratio test (Dobson 2002) to test this significance (ANOVA with argument “*Chisq*”). We calculated the *p* values for the individual predictors based on likelihood ratio tests between the full and the null model using the R function “*drop1*” (Barr et al. 2013). For GLMM_1_ and GLMM_3_, the response variable was binary; hence we used a binomial error distribution. For GLMM_2_, we log_10_-transformed the daily proportion of time spent grooming to reach a normal distribution after verifying the distribution and homogeneity of the residuals by the visual inspection of the qqplot and plotting the residuals against the fitted values (Estienne et al. 2017). For multinomial predictors with a significant main effect, we used a multiple contrast package (*multcomp*) to perform all pairwise comparisons for each bonding level with the Tukey test (Bretz et al. 2010). In this case, the level of probability was adjusted according to the Bonferroni correction. The effect size was calculated via the package “*effects*”.

## Results

### Prediction 1: direct and indirect human disturbance

As concerns prediction 1a, we found that human direct disturbance was significantly more frequent for frequent crop users than for infrequent crop users (exact Mann–Whitney test: *N*_frequent_crop_users_ = 8, *N*_infrequent_crop_users_ = 5, *U* = 4.000, *Z* = −2.342, *P* = 0.019).

**Fig. 2:**
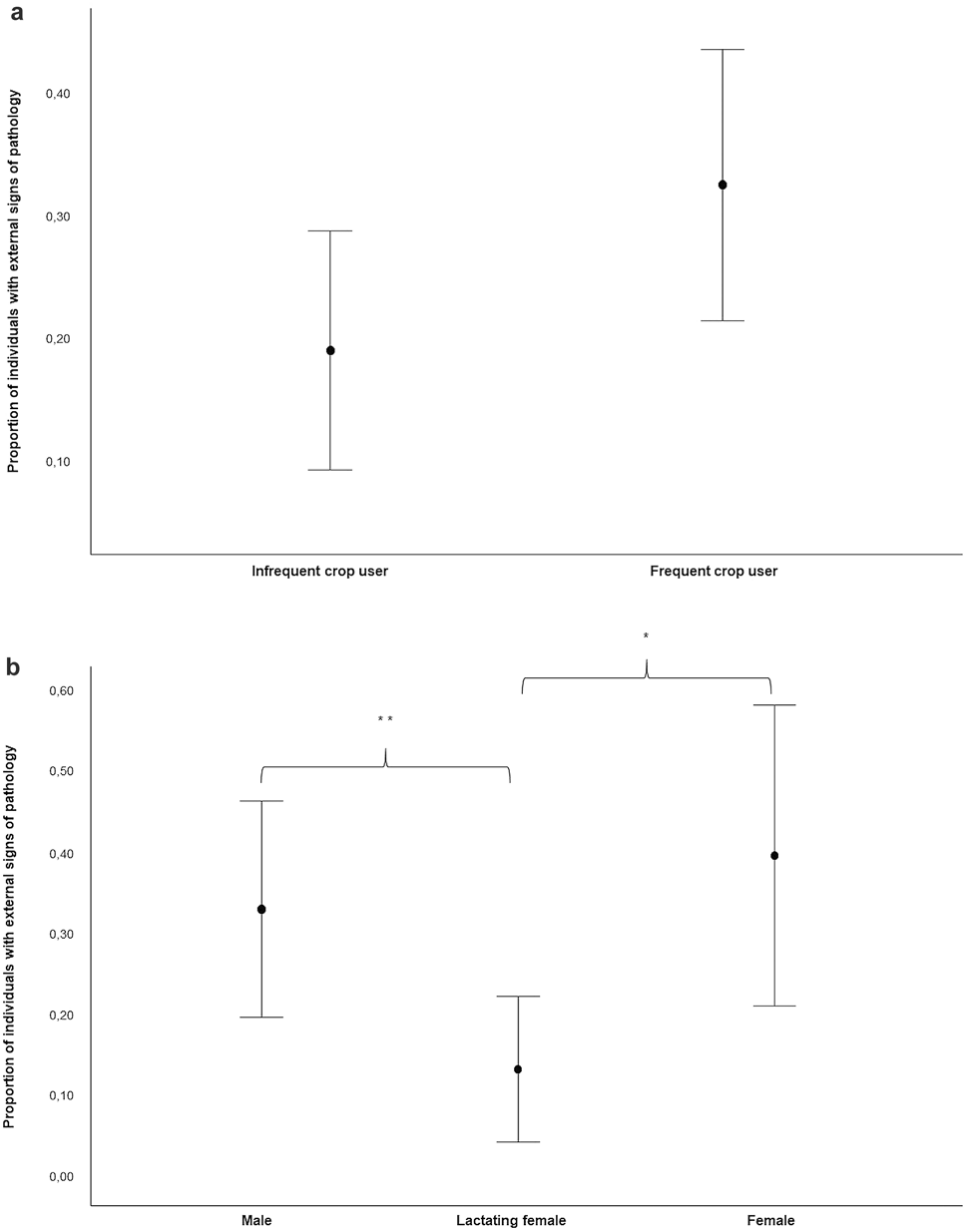
**a** Proportion of individuals with external signs of pathology in infrequent and frequent crop users (GLMM_1_, *N* = 140, variable condition: *χ*^2^ = 2.198, *P* = 0.028; full results: Table 1); **b** proportion of individuals with external signs of pathology in non-lactating females, lactating females, and males (GLMM_1_, *N* = 140, variable condition: *χ*^2^ = −2.417, *P* = 0.016; full results: Table 1). Mean (circle) and 95% confidence (bars) are indicated. *P < 0.05 and **P < 0.01

Via GLMM_1_, we tested the variables that potentially affected the presence of external signs of pathology (target variable; *N* = 140 cases) (prediction 1b). The full model differed significantly from the null model (likelihood ratio test: *χ*^2^ = 18.102, *df* = 5, *P* = 0.003). There was a small to moderate but significant effect of the variable group category (frequent crop users/infrequent crop users; effect size = 0.334; *P* = 0.028; Table 1) and sex (effect size = 0.398; *P* = 0.016; Table 1) on the target variable. Moreover, a trend of significance was observed for the variable age (effect size = 0.272; *P* = 0.055; Table 1). In particular, the prevalence of external signs of pathology was highest in the frequent crop users, and among adults it was lower in lactating females than in males and non-lactating females (Fig. 2a and b; Table 1; Tukey test; non-lactating females vs. lactating females, Est = 1.695; SE = 0.587, *P* = 0.011; lactating females vs. males, Est = −1.392, SE = 0.576, *P* = 0.041; non-lactating females vs. males, Est = 0.304, SE = 0.543, *P* = 0.842).

**Table 1:** Results of GLMMs

Predictors	Estimates	SEM	CI_95_	*χ*^2^	*P*
GLMM_1_ presence of external signs of pathology (*N* = 140) (group identity was included as random factor)					
(Intercept)^a^	0.591	0.892	−1.156, 2.338	a	a
Sex (lactating females)^b^	−1.392	0.576	−2.520, −0.263	−2.417	0.016
Sex (non-lactating females)^b^	0.304	0.543	−0.761, 1.368	0.559	0.576
Age class (immature)^b^	−1.044	0.545	−2.112, 0.024	−1.915	0.055
Group_category (frequent crop users)^b^	1.189	0.541	0.129, 2.249	2.198	0.028
OMU size	−0.097	0.062	−0.217, 0.024	−1.573	0.116
GLMM_2_ time spent daily in grooming interactions (*N* = 95) (dyad and unit identity were included as random factors)					
(Intercept)^a^	−2.167	0.589	−3.321, −1.011	a	a
Sex_actor (female)^b^	−0.419	0.331	−1.067, 0.229	−1.265	0.210
Sex_receiver (female)^b^	−0.119	0.271	−0.651, 0.412	−0.440	0.662
Age class_actor (adult)^b^	0.538	0.445	−0.333, 1.410	1.210	0.230
Age class_receiver (adult)^b^	−0.224	0.313	−0.388, 0.837	0.717	0.482
Area (crop)^b^	−0.590	0.225	−1.031, −0.148	−2.622	0.010
Crop_users (frequent crop users)^b^	−0.377	0.256	−0.878, 0.124	−1.474	0.146
Group_type (AMU)^b^	0.116	0.468	−0.800, 1.032	0.248	0.805
Season (small rainy season)^b^	0.583	0.212	0.167, 0.999	2.047	0.007
GLMM_3_ intensity of aggression (*N* = 114) (dyad and OMU identity were included as random factors)					
(Intercept)^a^	0.512	0.564	−0.534, 1.557	a	a
OMU (inter-OMU)^b^	1.143	0.779	−0.383, 2.669	1.467	0.142
Sex_dyad (different sex)^b^	−0.017	0.486	−0.969, 0.935	−1.035	0.972
Area (crop)^b^	−1.478	0.534	−2.524, −0.432	−2.770	0.006
Age_dyad (different age)^b^	−0.588	0.470	−0.333, 1.508	1.251	0.211
Crop_user_dyad (different crop user frequency)^b^	0.720	0.875	−2.435, 0.995	0.823	0.411
Season (small rainy season)^b^	−0.731	0.541	−1.791, 0.329	−1.352	0.176

^a^Not shown as not having a meaningful interpretation

^b^These predictors were dummy-coded, with the reference categories as follows: GLMM_1_–Sex: “male”; Age class: “adult”; Group_category: “infrequent crop users”; GLMM_2_–Sex actor/receiver: “male”; Age class actor/receiver: “immature”; Area: “pasture”; Crop users: “infrequent crop users”; Group_type: “OMU”; Season: “dry season”; GLMM_3_–OMU: “intra-OMU”; Sex_dyad: “same sex”; Area: “pasture”; Age dyad: “same age”; Crop user dyad: “same crop user frequency”; Season: “dry season”

In the following analysis, we checked for differences in the number of parasitic elements/g found in the feces of frequent and infrequent crop users (prediction 1c). In the fecal samples of both frequent and infrequent crop users we found *Giardia intestinalis* (detected for the first time in a wild gelada population; mean ± SE_infrequent_users_ = 1480.00 ± 851.66; mean ± SE_frequent_users_ = 386.38 ± SE 198.37), *Ancylostomatidae* (mean ± SE_infrequent_users_ = 231.45 ± 63.75; mean ± SE_frequent_users_ = 249.68 ± 67.47), *Chilomastix* spp*.* (mean ± SE_infrequent_users_ = 36.14 ± 10.43; mean ± SE_frequent_users_ = 30.32 ± 19.08), *Endolimax nana* (mean ± SE_infrequent_users_ = 22.21 ± 6.05; mean ± SE_frequent_users_ = 18.63 ± 3.09), and *Entamoeba histolytica/dispar* (mean ± SE_infrequent_users_ = 1.31 ± 0.73; mean ± SE_frequent_users_ 21.47 ± 12.99). We found that the number of parasitic elements/g of *Entamoeba histolytica/dispar* was significantly greater in frequent crop users compared to infrequent crop users (exact Mann–Whitney: *N*_infrequent_users_ = 29, *N*_frequent_users_ = 19, *U* = 128.50, *P* < 0.001). There was, however, no significant difference between frequent and infrequent crop users in the number of parasitic elements/g (i.e. egg/larva/oocyst/cyst) of *Ancylostomatidae, Chilomastix* spp*., Endolimax nana* or *Giardia intestinalis* (exact Mann–Whitney: *N*_infrequent_users_ = 29, *N*_frequent_users_ = 19; *Ancylostomatidae*: *U* = 262.00, *P* = 0.776; *Chilomastix* spp*.*: *U* = 223.50, *P* = 0.207; *Endolimax nana*: *U* = 241.00, *P* = 0.443; *Giardia intestinalis*: *U* = 243.50; *P* = 0.500).

### Prediction 2: impact of crop and pasture areas on social behavior

Geladas foraged significantly less in the crop areas in comparison to pasture (Wilcoxon signed-rank test via Monte Carlo randomization: *N*_days_ = 48, z = −4.544, *P* < 0.001; mean ± SE_crop_ = 0.306 ± 0.058; mean ± SE_pasture_ = 0.760 ± 0.035).

**Fig. 3:**
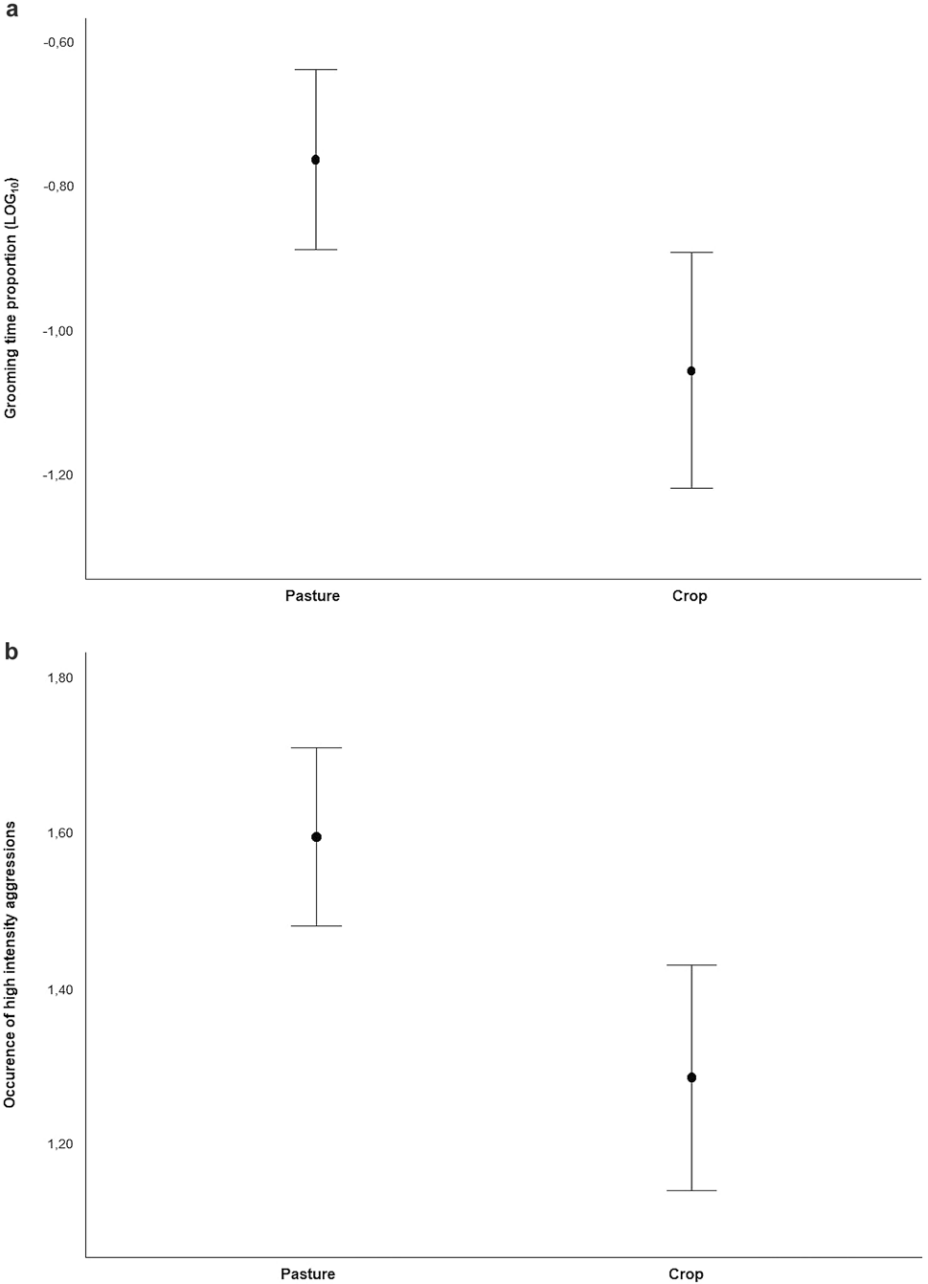
**a** Daily proportion of time spent in grooming interactions in pasture and crop area (GLMM_2_, *N* = 95, *t* value = −2.622, *P* = 0.010; full results: Table 1); **b** proportion of high-intensity aggression between pasture and crop area (GLMM_3_, *N* = 114, variable condition: *χ*^2^ = −2.770, *P* = 0.006; full results: Table 1). Mean (circle) and 95% confidence (bars) are indicated

In GLMM_2_, we tested what variables potentially affected the time that the dyads spent grooming on a daily basis (*N*_dyads_ = 95) (prediction 2a). The full model differed significantly from the null model (likelihood ratio test: χ^2^ = 19.748, *df* = 8, *P* = 0.011). Gelada dyads spent significantly more time grooming in the pasture than in the crop area (Fig. 3a; Table 1) and during the small rainy season than during the dry season (Table 1), with both variables showing a strong effect (absolute effect size > 1).

In GLMM_3_, we tested what variables potentially affected the intensity of aggression (*N*_aggressive_events_ = 114) (prediction 2b). The full model differed significantly from the null model (likelihood ratio test: *χ*^2^ = 15.723, *df* = 6, *P* = 0.015). The variable area (crop/pasture) had a moderate to large significant main effect on agonistic encounters (effect size = 0.616; Table 1). In particular, geladas engaged in more intense aggressive events when they were in the pasture area than when they were in the crop area (Fig. 3b; Table 1).

## Discussion

### Direct and indirect human disturbance

Our results are consistent with the hypothesis that crop area can be challenging to wild geladas, because frequent crop users were more exposed to direct human disturbance (in line with prediction 1a) and a waterborne parasite (i.e. *Entamoeba histolytica/dispar*; in partial agreement with prediction 1c), and showed more signs of external pathologies (i.e. alopecia and abnormal swelling), in line with prediction 1b (Table 1; Fig. 2a, 1b).

According to previous studies on geladas and other primates, the observed external signs of pathology were compatible with the presence of ectoparasites (i.e. alopecia) or endoparasites (i.e. abnormal swelling) possibly shared with livestock and humans (Toft 1986; Schneider-Crease et al. 2017). Throat swelling and alopecia may also be symptoms of iodine deficiency, which is common in the human population living in the Amhara region of Ethiopia, where this study took place (Abuye and Berhane 2007). These two pathology signs have also been observed in captive geladas (Borst et al. 1972). Similar symptoms may be caused by thyroid-disrupting chemical contaminants, including those used in agriculture (Maliszewska-Kordybach and Smreczak 1998; Rolland 2000). In particular, the 2,4-dichlorophenoxyacetic acid used in the study area as herbicide (pers. obs.) has been reported to cause tumors in humans (Anthony and Saleh 2013) and alopecia in dogs (Charles et al. 1996). Currently there is a lack of information on whether iodine deficiency and the abovementioned herbicide are also responsible for external signs of pathology in geladas. Hence, the causality of alopecia remains unclear, whereas swelling is most likely the result of infection with *Taenia* spp. (*Taenia serialis* in wild geladas) reported for other gelada populations, with canids being the primary host of this tapeworm (Ohsawa and Dunbar 1984; Nguyen et al. 2015; Schneider-Crease et al. 2017). In the study area, domestic dogs were present mostly around houses and crops, but both domestic and stray dogs may have roamed crop- and pastureland, which might explain why the area had only a small to medium effect on the presence of external signs of pathology. Even if we cannot confirm the presence of *Taenia serialis* in our study population from a biological point of view (to confirm the presence of this parasite, it is necessary to analyze urine samples; Schneider-Crease et al. 2017), the presence of abnormal swelling may be a predictor of the presence of this parasite. Indeed, *Taenia serialis* develops in the hypodermal musculature, causing abnormal swelling, and at the end of its development process the parasite perforates the skin and exits, causing suppurating masses (Ohsawa 1979). Once all the mass is purged, the swelling disappears (Dunbar 1980). On the other hand, the fact that apart from parasites, other factors specifically associated with farming may be linked to abnormal swelling and alopecia might explain why the effect of the area on the presence of external signs of pathologies was nevertheless significant. A diagnosis could not be performed on biological samples; therefore none of these possibilities can be ruled out.

The fact that the external signs of pathology were significantly more frequent in non-lactating adult females than in lactating females (Fig. 2b) might be related to the immunological properties of oxytocin, produced during lactation to regulate milk production (Wang 2016). On the contrary, testosterone in males can weaken the immune system, potentially explaining the more frequent signs of pathology in adult males than adult females (Roberts et al. 2004; Weisman et al. 2014; Muller 2017). Another, nonexclusive explanation is that females with abnormal swelling may be in poorer health conditions and therefore less able to reproduce (Nguyen et al. 2015). The effect of sex, although significant, was small to moderate, possibly because various factors, together or separately, can cause alopecia and abnormal swelling (including parasites and chemical pollutants as described above).

The trend observed in the increase of the external signs of pathology in adults is in line with previous studies on geladas (Nguyen et al. 2015; Schneider-Crease et al. 2017). The higher frequency of these signs in adult than in immature subjects could be related to parasite accumulation and/or higher stress levels. Adult subjects are more affected by social and environmental stress than immatures, causing a decrease in their immune system and making them more susceptible to parasitic infections (Muehlenbein and Bribiescas 2005).

We also found the presence of a wide range of gastrointestinal parasites (Nematoda and Protozoa) in gelada fecal samples. Most of the parasites detected showed no differences between frequent and infrequent crop users. However, we found that *Entamoeba histolytica/dispar* was highest in the feces of the frequent crop users. This result may be linked to the especially high contamination levels by *E. histolytica* reported for the Amhara region around human settlements, compared to other regions of Ethiopia (Aiemjoy et al. 2017; Zemene and Shiferaw 2018). In addition to indirect human disturbance (prevalence of external pathology signs and highest fecal parasite load), direct human disturbance was also high in the crop area. As a matter of fact, in the crop area, geladas were most likely to be chased away. This may have negative implications for gelada welfare. In other species, for example, it has been found that human–primate interactions (or even proximity) can be detrimental to health due to decreased feeding efficiency (related to increased vigilance for human aggression) and increased stress levels related to interactions with or threats by humans (Behie et al. 2010; Maréchal et al. 2011; Jaimez et al. 2012; Shutt et al. 2014; Chowdhury et al. 2020).

In summary, the first block of results suggests that agricultural activities close to human settlements can have a strong impact on wild gelada health. Frequenting agricultural areas may allow access to concentrated, high-quality resources (Strum 1994; Osborn and Hill 2005; Riley et al. 2013), but in the long term, crop foraging can have negative consequences on gelada health due to both direct and indirect disturbance. Further analyses on fecal samples collected from individuals showing external signs of pathologies could enable the identification of the possible direct link between the observed signs and parasite infections.

### Differences in social behavior: crop versus pasture area

The time spent grooming was higher in the pasture than in the crop area (in line with prediction 2a; Table 1; Fig. 3a). However, contrary to our prediction 2b, aggressive events were more intense in the pasture than in the crop area (Table 1; Fig. 3b).

Relatively few studies have investigated how human–primate interfaces can impact social relationships in primates, and the results of these studies are conflicting. For example, in contrast to our findings, studies on pygmy marmosets (*Cebuella pygmaea*: de la Torre et al. 2000) and on commensal macaque and baboon populations (Jaman and Huffman 2013) revealed that groups living in close proximity to human villages spent more time grooming than the groups living in the countryside. On the other hand, other studies are consistent with our results. A previous report on *Macaca sylvanus* (Majolo et al. 2013) described a decrease in grooming inside tourist areas. In a population of bonnet macaques (*Macaca radiata*), the individuals that interacted more frequently with humans showed a greater tendency to monitor human activity and a decrease in grooming (Balasubramaniam et al. 2020). A recent study found that despite a positive relationship between the value of resources and the time spent in affiliative behavior, human interference had negative effects on grooming (Thatcher et al. 2019). The apparently divergent effects of human presence on social grooming may depend on the extent to which animals frequent anthropized areas, how far they are from human-monitored edges (e.g. Priston et al. 2012), whether they are regularly or occasionally exposed to human disturbance, and the type of disturbance. The fact that the area had a small though significant effect on the time spent grooming highlights the importance of grooming in geladas, because a certain level of this behavior is maintained in challenging locations (crop) as well. Indeed, grooming in geladas is used to preserve and reinforce social relationships (Mancini and Palagi 2009), as in all the other cercopithecine species (Dunbar 1991). Similar to previous reports on geladas and other primate species (Lee 1984; Norscia et al. 2006; Yazezew et al. 2020), we found that the daily time spent grooming was higher in the rainy than in the dry season. During the dry season, food resources are normally distributed in more dispersed patches, and primates allocate more time to food search than to social interaction (Dunbar 1992).

Acute anxiety due to transient challenging situations can lead to a reduction in social behavior, including both affiliation and aggression (Kalin and Shelton 2003). The latter situation can apply to our study animals, which did not permanently live in proximity to human settlements: during the day, geladas came from the cliffs, entered the crop area to find better resources, acquired them from agricultural fields when possible, and then left. Indeed, geladas foraged significantly less in the crop than in the pasture area during the study period. This issue, along with other factors discussed below, can explain why in our study the aggressive events were less intense in the crop area, contrary to expectations. It has indeed been observed that the increased competition over high-value resources available in small patches can lead to increased conflicts in primate groups (*Macaca mulatta*: Southwick et al. 1976; *Papio anubis*: Wrangham 1974; *Pan troglodytes*: Wittig and Boesch 2003). However, stressful or threatening conditions can lead to behavioral suppression (Kalin and Shelton 2003), also in the case of human presence (Maréchal et al. 2011; Muehlenbein et al. 2012). Behavioral suppression, including reduced aggression, can be a strategy to avoid conflicts when they are too dangerous (e.g. crowded conditions with limited possibility of escape) or to reduce detection probability (Judge and de Waal 1993; le Roux et al. 2013). Considering that the study animals were not under crowded conditions (the groups frequenting the crop were not all present at the same time on the plateau), the second explanation is the most likely. Consistently, a previous study found that baboons (*Papio anubis*) can increase vigilance and reduce vocalizations to forage in crop fields (Warren 2009). A previous study (le Roux et al. 2013) found that a concealing behavior is present in geladas, which show vocal suppression during extra-pair copulations in order to reduce the risk of potential aggression by the dominant male. Reducing social affiliation and aggression intensity may allow animals to focus on food provisioning, spend less time in the crop area (than in the pasture area), and decrease the probability of being detected. The area had a moderate to strong effect on aggression intensity, probably because of the importance of reducing risk while acquiring high-quality resources. Hence, when frequenting the area most exposed to human disturbance, geladas reduced their social behavior to possibly maximize provisioning and minimize detection risk.

In conclusion, this work provides a novel assessment of direct and indirect human impact on a wild population of *Theropithecus gelada* living in an unprotected area, in terms of both health status and social behavior. From a conservation point of view, our results highlight that in order to properly assess animal welfare in the wild, it is important to consider not only demographic data but also the impact that human activities can have on health and, importantly, on social interactions between subjects. Further parasitological analyses and seasonal data across the years and in different areas are necessary to fully clarify the repercussions of human disturbance on the welfare and social dynamics of wild geladas living in unprotected areas.

## Supplementary Information

Below is the link to the electronic supplementary material.

Supplementary file1 (DOCX 15 KB)

Supplementary file2 *MPEG-1*: Human chasing a group of gelada with a stick. Video by Ivan Norscia (MP4 726 KB)

Supplementary file3 *MPEG-2*: Inter-OMU aggression in the pasture area. Video by Ivan Norscia (MP4 13991 KB)

Supplementary file4 Figure S1: GPS point distribution of (i) infrequent crop users in pasture (a) and crop (c) areas; (ii) frequent crop users in pasture (b) and crop (d) areas. GPS points refer to both OMUs (referring to the alpha-male) and AMUs positions (referring to the male of the group closest to the observer). During the study period, 1697 GPS points were collected (1327 in the pasture area and 370 in the crop area). (TIF 8179 KB)

Supplementary file5 Figure S2: Rifle cartridge collected on the Kundi plateau, used to chase geladas away from crops. Photo by Ivan Norscia(TIF 13662 KB)

Supplementary file6 (DOCX 16 KB)

## Data Availability

The study data are available from the corresponding author upon reasonable request.
