# Supplementary material for: Wild geladas (Theropithecus gelada) in crops—more than in pasture areas—reduce aggression and affiliation
Source: Primates. 2021 Jun 1;62(4):571–84. doi: 10.1007/s10329-021-00916-8 (PMC8225520; doi:10.1007/s10329-021-00916-8)
Supplement: Supplementary file 1 — Supplementary file1 (DOCX 15 KB) [file 10329_2021_916_MOESM1_ESM.docx]

Primates

**Wild geladas (*Theropithecus gelada*) in crops – more than in pasture areas – reduce aggression and affiliation**

Marta Caselli^1^, Anna Zanoli^1^, Carlo Dagradi^1^, Alessandro Gallo^1^, Dereje Yazezew^2^, Abebe Tadesse^2^, Michele Capasso^3^, Davide Ianniello^3^, Laura Rinaldi^3^, Elisabetta Palagi^4,5,*^, Ivan Norscia^1,5,*^

^1^University of Torino, Department of Life Sciences and Systems Biology

^2^Debre Behran University, Department of Biology

^3^University of Napoli Federico II, Department of Veterinary Medicine and Animal Production

^4^University of Pisa, Department of Biology, Unit of Ethology

^5^University of Pisa, Natural History Museum

*Shared corresponding and senior authors: Ivan Norscia^1,5^, Department of Life Sciences and Systems Biology, University of Torino, via Accademia Albertina 13, 10123, Torino, Italy. Email: [ivan.norscia@unito.it](about:blank) Tel: +39 011 6704547

Elisabetta Palagi^4,5^, Unit of Ethology, Department of Biology, University of Pisa, via Volta 6, 56126, Pisa, Italy. Email: [elisabetta.palagi@unipi.it](about:blank) Tel: +39 050 2211385

**Appendix S1 – Additional information**

**Additional information on study site**

Temperature and humidity were measured and recorded for every observation day (via Weather Station Thomson 513630). The minimum and maximum mean temperatures were 7.13 ± SE 0.13 °C and 12.46 ± SE 0.33 °C, respectively. The minimum and maximum mean humidity values were 72.88 ± SE 2.29% and 93.78 ± SE 0.93%, respectively. During the study months, seasonality on the Kundi plateau was characterized by the following periods: dry season (from December to March) and small rainy season (from April to May).

Crop areas mainly consisted in cultivations of cereal tef (*Eragrostis tef*) and small crops of potatoes (*Solanum tuberosum*). Pasture areas were dominated by graminaceous species, and particularly *Festuca* spp. The habitat falls into the Afro-alpine ecosystem and, except for cultivated plants, the only detected exotic species was *Eucaliptus* sp., on the cliffs. In both crop and pasture areas, cattle, sheep, goats, horses, and donkeys were present, but horses and donkeys usually stayed close to the human settlements. Dogs were also present in the crop area. Besides geladas, the medium-large, wild mammals present in the area included the primate *Colobus guereza*, the carnivore *Crocuta crocuta*, and the ungulates *Oreotragus oreotragus*, *Tragelaphus* spp., *Ourebia ourebi*. Other species are reported in the area but were not seen during our study (i.e. carnivores *Otocyon megalotis* and *Panthera pardus;* Yalden et al. 1996).

**Additional information on study population**

The mean number of individuals per OMU varied both for females and males. The mean number of males varied due to the presence of peripheral males in some OMUs: adult males = 1.286 ± SE 0.125, adult females = 5.643 ± SE 0.372, sub-adults = 4.214 ± SE 0.505, juveniles = 2.429 ± SE 0.552, infants = 4.643 ± SE 0.325 (late infants = 2.214 ± SE 0.334; early infants =1.500 ± SE 0.272; black infants = 0.929 SE ± 0.304). The mean number of adult males per AMU was 5.000 ± SE 2.000.

**Additional information on pathological individuals**

The mean number of individuals with external signs of pathology in infrequent or frequent crop user groups: i) adult males with swelling 0.375 ± SE 0.263 and 0.625 ± SE 0.263; ii) adult males with both alopecia and swelling 0.000 ± SE 0.000 and 0.125 ± SE 0.125; iii) adult females with alopecia 0.000 ± SE 0.000 and 0.125 ± SE 0.125; iv) adult females with swelling 0.625 ± SE 0.375 and 1.250 ± SE 0.628; v) adult females with both alopecia and swelling 0.250 ± SE 0.164 and 0.250 ± SE 0.164; vi) immatures with swelling 0.375 ± SE 0.183 and 0.625 ± SE 0.263 (the categories not reported did not present individuals with external signs of pathology). In total, 15% of individuals showed one or more of signs of external pathology.
