# Supplementary figures and images for: Wild geladas (Theropithecus gelada) in crops—more than in pasture areas—reduce aggression and affiliation
Source: Primates. 2021 Jun 1;62(4):571–84. doi: 10.1007/s10329-021-00916-8 (PMC8225520; doi:10.1007/s10329-021-00916-8)

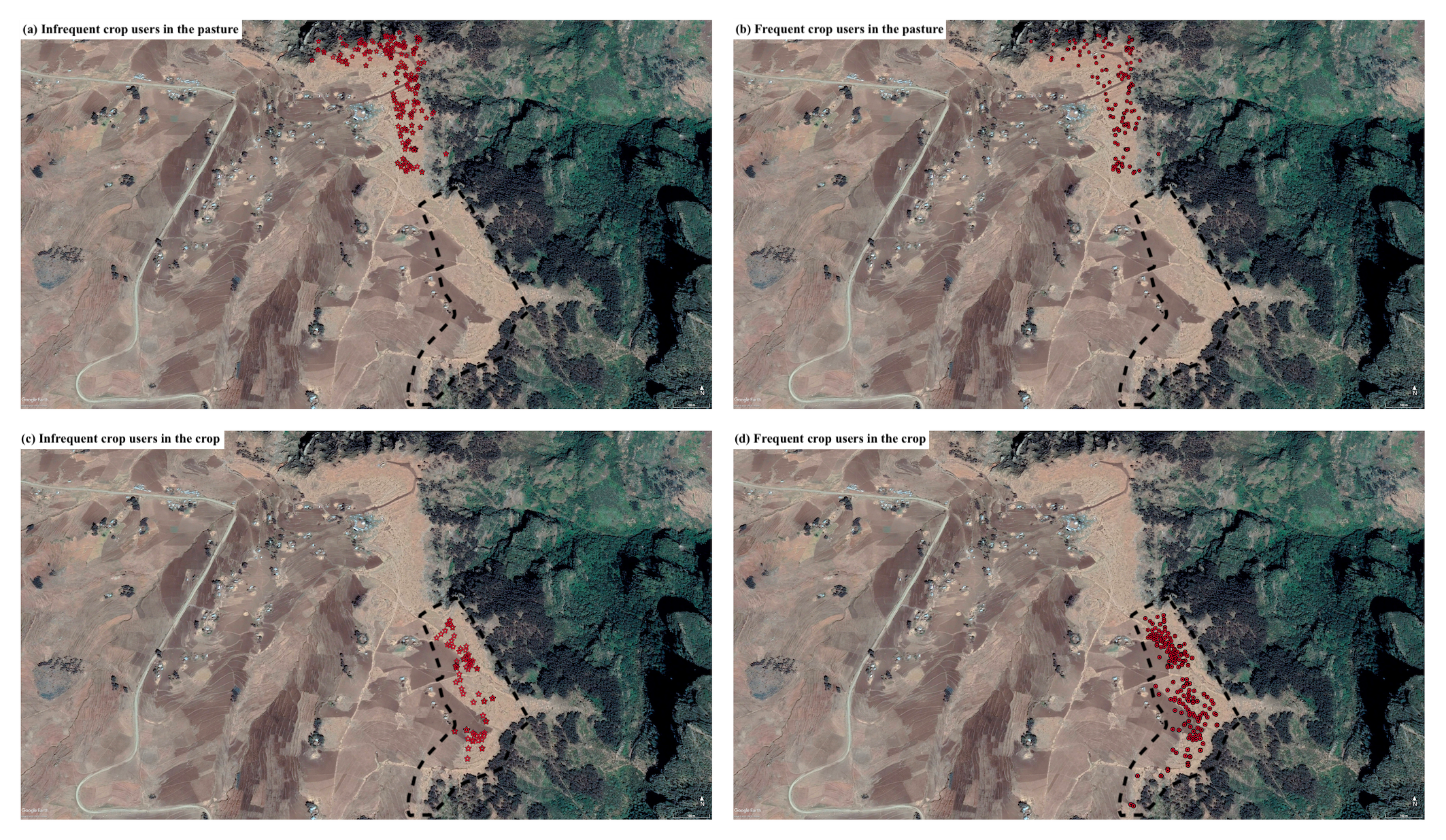

Supplement: Supplementary file 4 — Supplementary file4 Figure S1: GPS point distribution of (i) infrequent crop users in pasture (a) and crop (c) areas; (ii) frequent crop users in pasture (b) and crop (d) areas. GPS points refer to both OMUs (referring to the alpha-male) and AMUs positions (referring to the male of the group closest to the observer). During the study period, 1697 GPS points were collected (1327 in the pasture area and 370 in the crop area). (TIF 8179 KB) [file 10329_2021_916_MOESM4_ESM.tif]

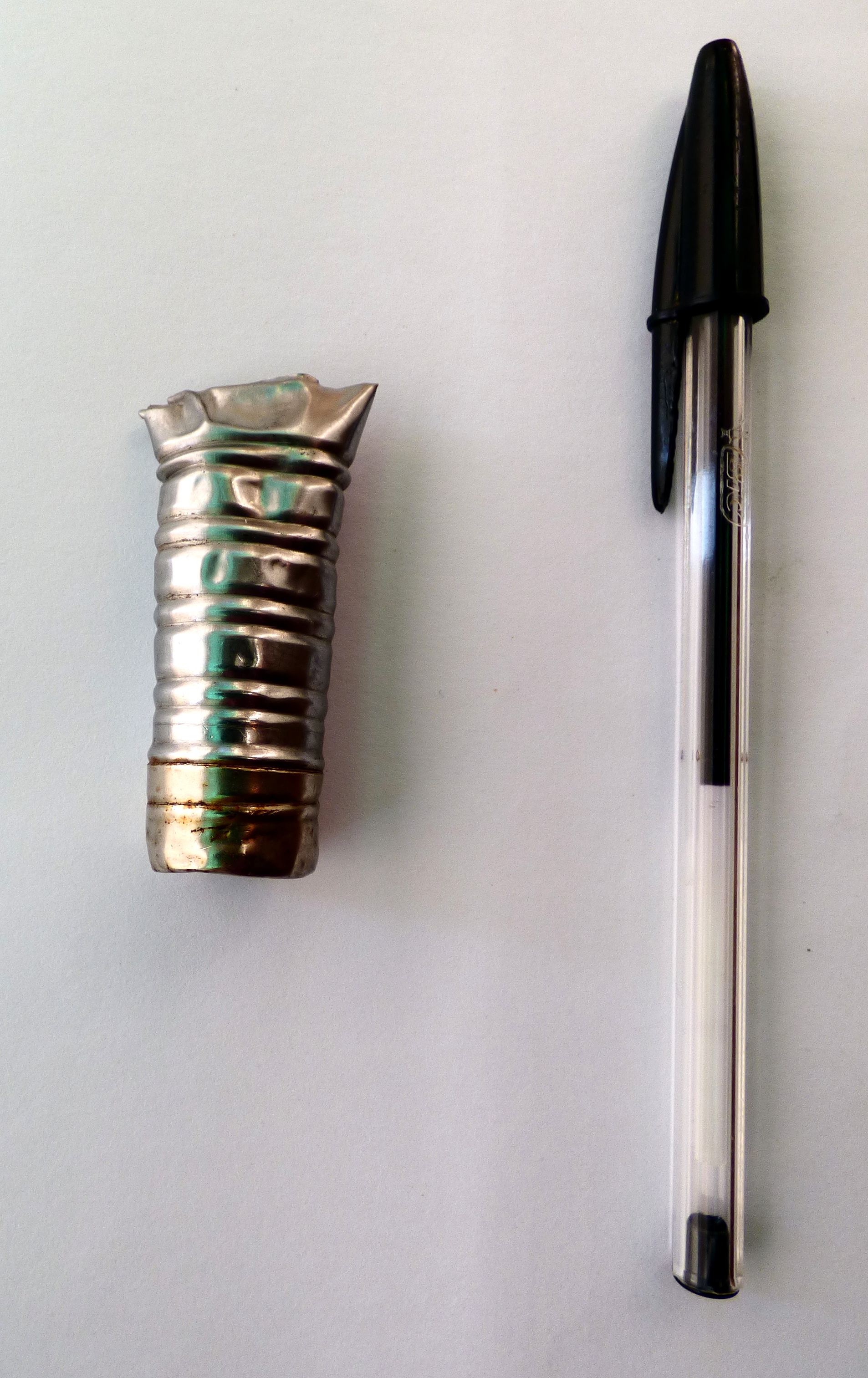

Supplement: Supplementary file 5 — Supplementary file5 Figure S2: Rifle cartridge collected on the Kundi plateau, used to chase geladas away from crops. Photo by Ivan Norscia(TIF 13662 KB) [file 10329_2021_916_MOESM5_ESM.tif]
