# Supplementary material for: Wild geladas (Theropithecus gelada) in crops—more than in pasture areas—reduce aggression and affiliation
Source: Primates. 2021 Jun 1;62(4):571–84. doi: 10.1007/s10329-021-00916-8 (PMC8225520; doi:10.1007/s10329-021-00916-8)
Supplement: Supplementary file 6 — Supplementary file6 (DOCX 16 KB) [file 10329_2021_916_MOESM6_ESM.docx]

Primates

**Wild geladas (*Theropithecus gelada*) in crops – more than in pasture areas - reduce aggression and affiliation**

Marta Caselli^1^, Anna Zanoli^1^, Carlo Dagradi^1^, Alessandro Gallo^1^, Dereje Yazezew^2^, Abebe Tadesse^2^, Michele Capasso^3^, Davide Ianniello^3^, Laura Rinaldi^3^, Elisabetta Palagi^4,5,*^, Ivan Norscia^1,5,*^

^1^University of Torino, Department of Life Sciences and Systems Biology

^2^Debre Behran University, Department of Biology

^3^University of Napoli Federico II, Department of Veterinary Medicine and Animal Production

^4^University of Pisa, Department of Biology, Unit of Ethology

^5^University of Pisa, Natural History Museum

*Shared corresponding and senior authors: Ivan Norscia^1,5^, Department of Life Sciences and Systems Biology, University of Torino, via Accademia Albertina 13, 10123, Torino, Italy. Email: [ivan.norscia@unito.it](about:blank) Tel: +39 011 6704547

Elisabetta Palagi^4,5^, Unit of Ethology, Department of Biology, University of Pisa, via Volta 6, 56126, Pisa, Italy. Email: [elisabetta.palagi@unipi.it](about:blank) Tel: +39 050 2211385

**Supporting material - Tables**

*Table S1*

| Human disturbances | Description | Crop | Pasture | Direct | Indirect |
| --- | --- | --- | --- | --- | --- |
| Chasing | Humans drive off geladas by running after them | **+** | **-** | **✓** |  |
| Throwing stones/sticks | Humans launch stones or use sticks to displace geladas | **+** | **-** | **✓** |  |
| Livestock | Humans lead livestock towards geladas to displace them | **-** | **+** | **✓** |  |
| Dogs | Humans use dogs to scare geladas away | **+** | **-** | **✓** |  |
| Whip | Humans crack the whip to scare geladas | **+** | **-** | **✓** |  |
| Shooting | Humans shoot to keep geladas away from crops | **+** | **-** | **✓** |  |
| Parasites | Possible transmission of parasites between human/livestock and geladas suggested by certain parasites in geladas fecal samples (e.g *Entamoeaba histolityca* and *Giardia intestinalis*) and gelada abnormal swelling/alopecia (increased in crops) | + | - |  | **✓** |
| Chemical pollutants/agricultural substances | Observed use of detergents and 2,4D fertilizer in the study area. Possible health damages linked to the use of such substances | + | - |  | **✓** |

Description of the different types of human disturbance observed/hypothesized during the study period. + = this type of human disturbance is more frequent in this area than in the other; - = this type of human disturbance is less frequent in this area than in the other.

| OMU/AMU | Frequency of scan in the crop area | Classification |
| --- | --- | --- |
| OMU1 | 0.147 | Infrequent crop user |
| OMU2 | 0.016 | Infrequent crop user |
| OMU3 | 0.306 | Frequent crop user |
| OMU4 | 0.336 | Frequent crop user |
| OMU5 | 0.185 | Infrequent crop user |
| OMU6 | 0.164 | Infrequent crop user |
| OMU7 | 0.285 | Frequent crop user |
| OMU8 | 0.177 | Infrequent crop user |
| OMU9 | 0.108 | Infrequent crop user |
| OMU10 | 0.264 | Frequent crop user |
| OMU11 | 0.286 | Frequent crop user |
| OMU12 | 0.193 | Frequent crop user |
| OMU13 | 0.298 | Frequent crop user |
| OMU14 | 0.184 | Infrequent crop user |
| AMU1 | 0.554 | Frequent crop user |
| AMU2 | 0.000 | Infrequent crop user |

*Table S2*

The table shows the frequency in the use of the crop area. Frequency was calculated by considering the number of scans in which each group was inside the crop area normalized over the total of scans/group. We separated the groups into two categories (‘frequent crop users’ and ‘infrequent crop users’), depending on whether the frequencies fell above or below the median frequency (median = 0.189) of the proportion of scans/group recorded in crops.
